# Addition of Carbon to the Culture Medium Improves the Detection Efficiency of Aflatoxin Synthetic Fungi

**DOI:** 10.3390/toxins8110338

**Published:** 2016-11-15

**Authors:** Tadahiro Suzuki, Yumiko Iwahashi

**Affiliations:** 1 Division of Food Biotechnology, Food Research Institute, NARO, 2-1-12 Kannon-dai, Tsukuba, Ibaraki 305-8642, Japan; suzut@affrc.go.jp; 2 Division of Food Safety, Food Research Institute, NARO, 2-1-12 Kannon-dai, Tsukuba, Ibaraki 305-8642, Japan

**Keywords:** aflatoxin, cyclodextrin, ultraviolet, carbon, plate culture

## Abstract

Aflatoxin (AF) is a harmful secondary metabolite that is synthesized by the *Aspergillus* species. Although AF detection techniques have been developed, techniques for detection of AF synthetic fungi are still required. Techniques such as plate culture methods are continually being modified for this purpose. However, plate culture methods require refinement because they suffer from several issues. In this study, activated charcoal powder (carbon) was added to a culture medium containing cyclodextrin (CD) to enhance the contrast of fluorescence and improve the detection efficiency for AF synthetic fungi. Two culture media, potato dextrose agar and yeast extract sucrose agar, were investigated using both plate and liquid cultures. The final concentrations of CD and carbon in the media were 3 mg/mL and 0.3 mg/mL, respectively. Addition of carbon improved the visibility of fluorescence by attenuating approximately 30% of light scattering. Several fungi that could not be detected with only CD in the medium were detected with carbon addition. The carbon also facilitated fungal growth in the potato dextrose liquid medium. The results suggest that addition of carbon to media can enhance the observation of AF-derived fluorescence.

## 1. Introduction

Aflatoxin (AF), which is synthesized by secondary metabolism in fungi, is a highly carcinogenic natural compound. AF is synthesized by particular *Aspergillus* species, including *A. flavus*, *A. parasiticus*, *A. nomius*, and *A. bombysis*. Genetic investigations of the AF synthetic mechanism have shown AF synthetic fungi contain a specific gene cluster [1,2,3,4,5,6,7,8,9]. However, many non-toxigenic strains also have this gene cluster, although these clusters may have partial deletions and/or substitutions compared to those in AF synthetic fungi. Additionally, many brewery fungi, including *A. oryzae* or *A. sojae*, contain some of these genes. To discriminate AF synthetic strains from non-toxigenic strains, polymerase chain reaction studies that target AF gene clusters have been conducted, and these strategies are effective [10,11,12,13]. However, the primer sets sometimes miss strains that produce AF [8]. In their present state, genetics-based methods are not yet reliable for detection of AF synthetic fungi. Therefore, optical methods using plate cultures are still useful.

It is known that AF has four main subtypes, and can be classified into two groups (B and G). The type B AFs are AFB_1_ and AFB_2_, and the type G AFs are AFG_1_ and AFG_2_. On exposure to ultraviolet (UV) light at 365 nm, type B and type G AFs show blue and green fluorescence, respectively. Therefore, the simplest method to detect AF synthetic fungi is UV irradiation of a culture plate. However, the obtained fluorescence intensity is not very high, and it can be difficult to detect because of the low intensity. Cyclodextrin (CD) has been used to increase the detection efficiency of AF-derived fluorescence [14,15,16,17,18]. AF readily enters CD, which is composed of several d-glucose molecules and has a circular structure with hydrophobic groups on the inside. One recent report suggested the influence of CD on AF fluorescence could be attributed to the lost ability to bind to water molecules on formation of the inclusion compound [19]. Formation of a CD inclusion compound is thought to improve the fluorescence intensity, and this method has been applied to the detection of AF synthetic fungi in environmental samples [20,21]. CD is grouped into three main types according to the number of d-glucose molecules it contains. Alpha (α), beta (β), and gamma (γ) CD have six, seven, and eight-membered sugar rings, respectively. On binding with AF, αCD shows the highest fluorescence intensity among the CDs, and this is followed by βCD [22]. Meanwhile, addition of γCD to AF does not change the fluorescence intensity. Therefore, αCD is likely the most appropriate size for inclusion of AF, whereas γCD is not suitable. Methylated βCD (mβCD) has also been used [15,23]. However, addition of CD to a plate culture for detection of AF synthetic fungi occasionally yields incorrect results [24].

As an alternative, an *A. flavus parasiticus* agar (AFPA) plate has been developed that produces an orange color for positive colonies without the requirement for a light source [25,26]. A coconut agar plate that fluoresces on UV irradiation has also been used to confirm AF synthesis [27,28]. However, these techniques can produce false positives, such as color production from the antibiotic aspergillic acid [25]. Despite these drawbacks, the AFPA technique is suitable for initial screening because it can be used for a wide range of *Aspergillus* strains. The coconut agar plate technique is only as useful as the CD technique because the fluorescence intensity is not high. Alternatively, addition of ammonia gives a rose red color for the intermediate of AF [29]. The ammonia method can be used with any regent, and recently, it was reported that the addition of ammonia to media containing dichlorvos improved the color dramatically [30]. However, the plate cover needs to be removed temporarily to add ammonia for this method, which could potentially lead to spore drift. The ammonia method can also give false positives [24].

Compared with these other methods, the CD method has many positives and is more efficient. Therefore, improvement of the CD method should be investigated. Generally, an observation technique with transmitted UV light is used to detect fluorescent rings around fungal colony. However, the fluorescence signals are sometimes unclear [24], which can lead to incorrect results. Fluorescence from the back of the fungal colony can be obtained under reflected light conditions, and might be more suitable than transmitted UV light for detection. However, scattering of light sometimes leads to unsatisfactory results. Therefore, to improve the CD method, we initially focused on scattered light observed around fungal colonies. The level of scattering of incident light after UV irradiation from above is thought to be dependent on the smoothness of the surface of the culture medium. A method for attenuating the reflection of light without negatively affecting fungal growth is required.

In the present study, to improve the detection efficiency of AF synthetic fungi with the CD method, we added activated charcoal powder (carbon) to attenuate the reflection of light derived from the plate. To take advantage of the adsorption of AF by the carbon and the associated reduction in the risk for acute toxicity [31,32,33], the carbon was initially fixed in a solid medium in a plate.

## 2. Results

### 2.1. Changes in the Optical Characteristics of the Culture Medium with Addition of Carbon

**Figure 1:**
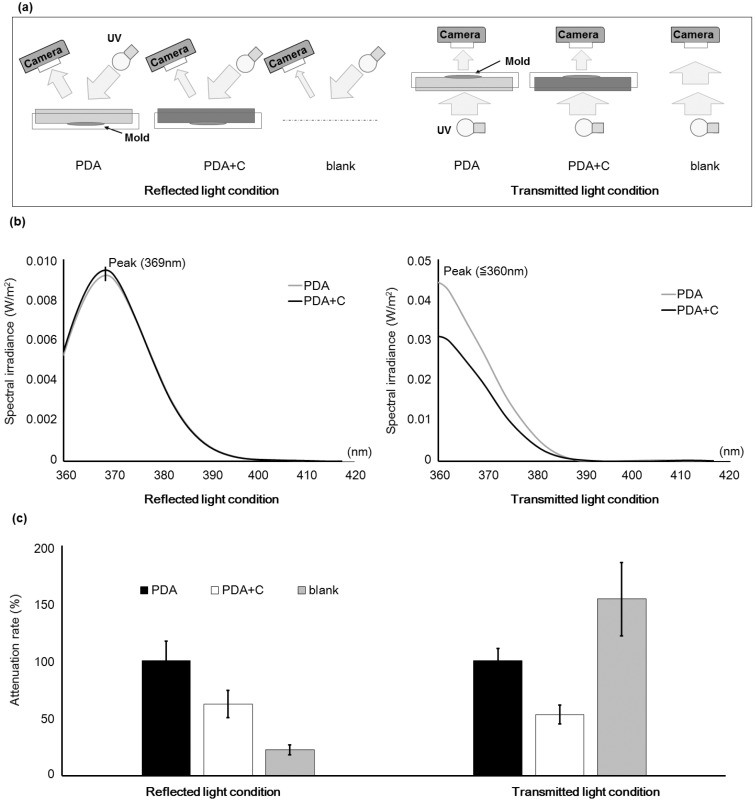
Testing conditions of UV irradiation and the effect of addition of carbon to the culture medium. (**a**) UV irradiation of the culture medium (potato dextrose agar (PDA)) with or without 0.3 mg/mL carbon, and detection of reflected and transmitted light. The blank experiment was conducted without a culture plate; (**b**) Wavelengths for reflected and transmitted light with and without carbon added to the PDA. Wavelengths less than 360 nm were not measured because of technical limitations; (**c**) Photon flux density (PFD) with and without addition of carbon to the PDA. Error bars show the standard deviation (*n* = 4).

After adding activated charcoal powder (carbon) to the potato dextrose agar (PDA) plate at a final concentration of 0.3 mg/mL, the wavelengths and intensities of reflected and transmitted light were observed from the surface under UV irradiation (Figure 1a). The results were used to investigate the influence of carbon on the surface of culture plate. Compared to incident light, the peak of the reflected light shifted to a longer wavelength, and that of the transmitted light did not change (Figure 1b). Addition of carbon to the plate decreased the values of photo flux density (PFD) for both reflected and transmitted light compared to the incident light (Figure 1c). Without the culture plate, the PFD of the reflected light decreased and that of transmitted light increased compared to the incident light.

#### 2.1.1. Changes in the Fluorescence with Different Culture Media

**Figure 2:**
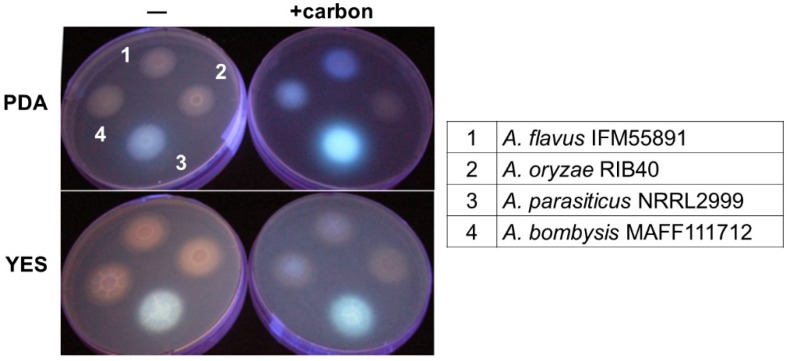
Changes in fluorescence with addition of 0.3 mg/mL carbon (− or + carbon) to the culture medium (potato dextrose agar (PDA) or yeast extract sucrose (YES) agar). The culture plates were incubated for two days and then irradiated with UV light (λ = 365 nm) from above.

The detection efficiencies for the culture plate method for discriminating AF synthetic fungi differ depending on the composition of the culture media and the incubation conditions [16,18,20,21]. In this study, the fluorescence intensities obtained with different *Aspergillus* strains on PDA and yeast extract sucrose (YES) agar plates were compared (Figure 2). When both αCD and carbon were added to these media, *A. flavus* IFM55891 and *A. bombysis* MAFF111712 could be detected, whereas they could not when only αCD was added. The fluorescence intensities obtained with the YES plate were lower than those obtained with the PDA plate. For *A. parasiticus* NRRL2999, the PDA plate with both carbon and αCD gave a high fluorescence intensity. The YES medium has a high sugar content, and fungal growth is faster in this medium than in PDA. However, these results show that the character of YES does not affect the fluorescence intensity.

#### 2.1.2. Effect of the Type of CD on the Fluorescence

**Figure 3:**
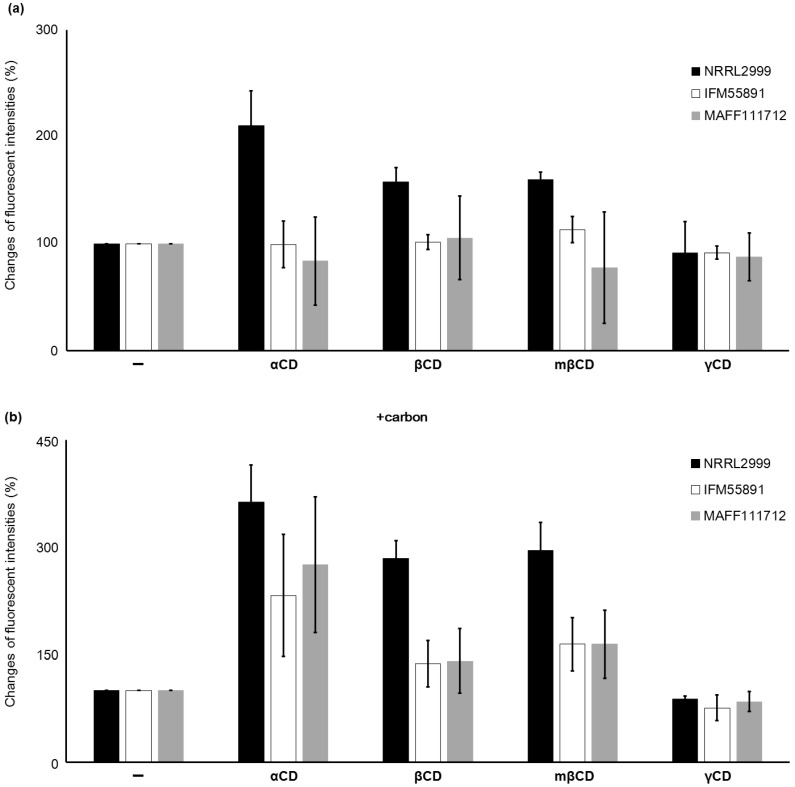
Changes in the fluorescence intensities with different types of cyclodextrin (CD) added to the medium without (**a**) or with (**b**) carbon. The change in fluorescence intensity for each condition was calculated relative to the fluorescence intensity of each sample without CD. The error bars indicate standard deviations (*n* = 3).

The level of inclusion of AF within CD depends on the structure of the specific CD, and αCD, which is a six-membered sugar ring, has the highest inclusion ability. This is followed by the seven-membered sugar ring βCD. The eight-membered sugar ring γCD does not enhance AF-derived fluorescence [22]. These differences suggest that αCD is the most useful CD for detection of AF. However, it is unclear whether the effect of αCD is dependent on the strain or species of AF synthetic fungi, or if carbon has an effect on the response obtained with CD. To investigate the influence of the type of CD on the fluorescence intensity, plates with and without carbon and different types of CD were compared (Figure 3). Without addition of carbon to the medium, the fluorescence intensity of *A. parasiticus* NRRL2999 with αCD was two times that obtained without αCD. Addition of βCD and mβCD also increased the fluorescence intensity, but not as much as αCD. With β CD, *A. parasiticus* NRRL2999 was the only *Aspergillus* strain to show an increase in fluorescence that could be detected by the naked eye. In the medium with added carbon, the fluorescence intensity for *A. parasiticus* NRRL2999 obtained with αCD was more than three times the fluorescence intensity obtained without αCD. Under the same conditions, the fluorescence intensity obtained with βCD was also higher than that obtained without βCD. Whereas no fluorescence was observed for either *A. flavus* IFM55891 or *A. bombysis* MAFF111712 in the medium without carbon, fluorescence was observed with carbon and some of the CDs. Addition of mβCD showed a slightly higher increases in the fluorescence intensities than βCD, but the differences were small.

#### 2.1.3. Correlation of the Color of Fluorescence with the *Aspergillus* Species and Strain

**Figure 4:**
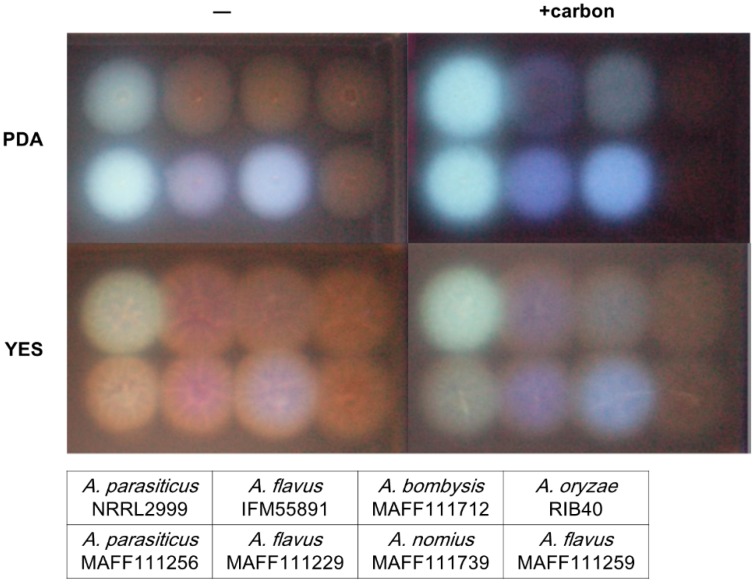
The relationship between the color of fluorescence and fungal species after 64 h of incubation on culture plates containing alpha CD (αCD). The plates were irradiated with UV light (λ = 365 nm) from above. The eight fluorescent spots in each image are for the colonies given in the text box below the figure.

The observed increases in the fluorescent intensity with addition of αCD and carbon to the PDA were useful to confirm the presence of AF synthetic fungi at an early stage of colony formation. At later stages (64 h incubation), different colors of fluorescence were observed (Figure 4). After culturing on PDA containing carbon, the color of fluorescence from *A. flavus* strains was different from that of the *A. parasiticus* strains. Colonies of *A. bombysis* and *A. nomius* also showed slightly different colors compared to the *A. parasiticus* strains. The YES plate showed stronger variation in the colors than the PDA plate, although the detection efficiency was inferior to the PDA plate. It should be noted that the strains *A. oryzae* RIB40 and *A. flavus* MAFF111259 do not produce AF.

### 2.2. Effect of the Addition of Carbon to a Liquid Culture

**Figure 5:**
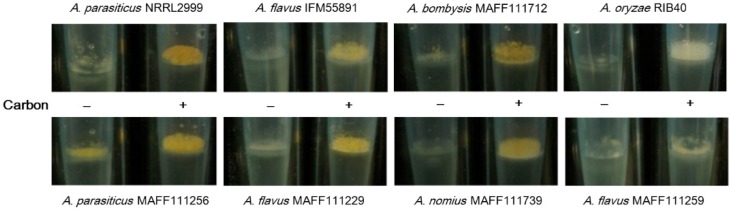
Colony formation on the surface of a liquid culture with (+) and without (−) carbon after incubation for seven days. Images are recorded from the side. The entire images of the cultures are given in Figure 9b.

**Figure 6:**
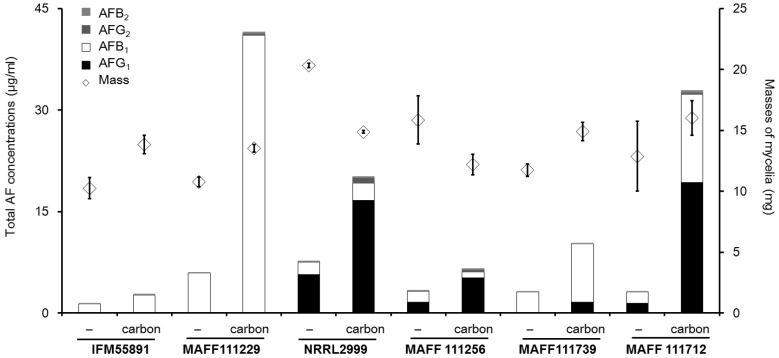
Total aflatoxin concentrations and fungal colony masses from liquid cultures with PD after incubation for seven days. The bar graphs showed combined data, and standard deviations are not indicated. The actual concentrations and standard deviations are given in Table 1. The error bars on the diamond symbols show the standard deviations (*n* = 4).

For the plate culture, the colony sizes and fungal growth did not appear to change even though addition of carbon to the medium enhanced the fluorescence intensity. Next, we investigated the application of this method to a liquid culture using a pipette tip culture method [34]. After incubation of the *Aspergillus* strains for seven days, thick colonies were observed on the surface of the potato dextrose (PD) medium (Figure 5). These fungal colonies weighed between 10 and 20 mg (Figure 6). For all strains except the *A. parasiticus* strains, the masses of the fungal colonies were higher in the medium containing carbon than in the medium without carbon. The *A. parasiticus* strains showed decreases in the fungal colony masses on addition of carbon to the medium.

Next, we measured the total AF concentration in the liquid culture media from the six AF synthetic strains. The total AF concentration ranged from 1 to 40 μg/mL, and the concentration was affected by both the *Aspergillus* species and composition of the medium (Figure 6). Regardless of the changes observed in fungal colony mass, the concentrations showed that AF synthesis increased in the medium with carbon compared to that without carbon. In the PD liquid culture, the masses of the fungal colonies of the AF synthetic strains changed by less than 40% on addition of carbon compared to those obtained without carbon. By contrast, the AF concentrations obtained for some strains with carbon were more than eight times those obtained without carbon (Table 1). The increases in the AFB_1_ and AFG_1_ concentrations were almost significant (*p* < 0.05), except for the AFB_1_ concentration from *A. flavus* IFM55891. The changes in the AFB_2_ and AFG_2_ concentrations were larger than those observed for AFB_1_ and AFG_1_. However, the absolute values were much lower than those for AFB_1_ or AFG_1_. Consequently, the changes in the AFB_2_ and AFG_2_ concentrations did not greatly contribute to the change in the total AF concentration. The concentrations for AFG_1_ from four of the strains were more than four times those obtained without carbon. However, only the concentration of AFB_1_ for *A. parasiticus* MAFF111256 showed a significant decrease (*p* < 0.05).

**Figure 7:**
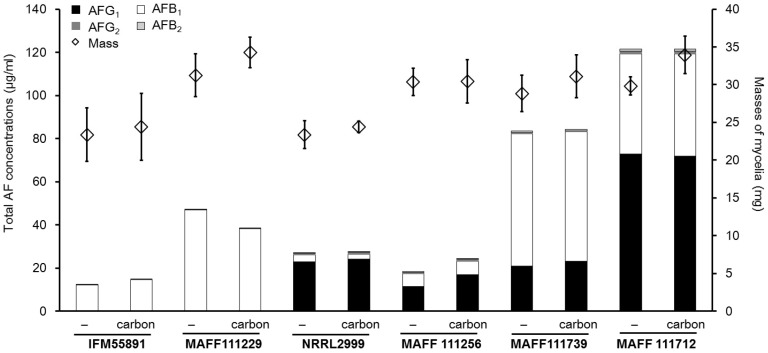
Total AF concentrations and masses of mycelia for liquid cultures using YES after incubation for four days. The bar graphs show combined data, and the standard deviations are not shown. The actual concentrations and standard deviations are given in Table 2. Bars on the diamond symbols indicate the standard deviations (*n* = 4).

After incubation for four days on YES, the fungal colonies were all well-developed regardless of the presence of carbon or not (Figure 7). The masses of all mycelia were greater than 20 mg, and the masses increased with addition of carbon for all strains except the *A. parasiticus* strains. The total concentrations of AF ranged from 10 to 120 μg/mL, and depended on the species and composition of the medium. However, it was difficult to evaluate whether these results were influenced by the presence of carbon in the medium. The increases in the concentrations compared to the medium without carbon were low (Table 2).

### 2.3. Factors not Related to the Changes in AF Concentrations

**Figure 8:**
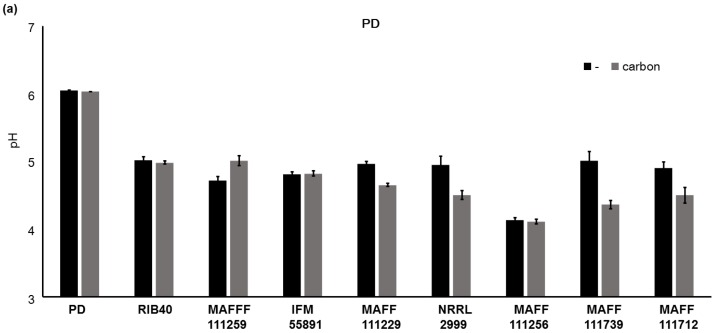

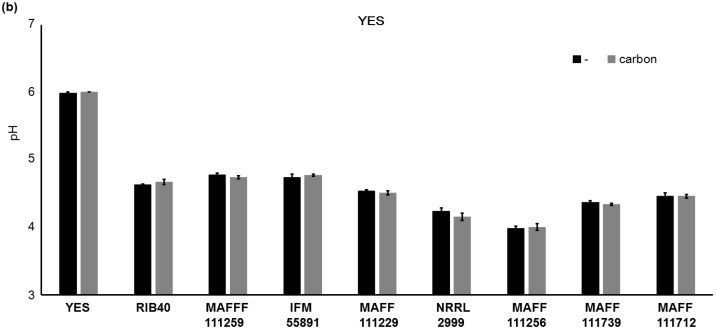
Changes in the pH of the media after the liquid culture test. In PD after seven days of incubation (**a**) and in YES after four days of incubation (**b**). The error bars show the standard deviations (*n* = 4).

We found that addition of carbon to the culture medium increased the fluorescence intensity, growth of the fungi, and AF synthesis. However, it is not clear why the addition of carbon to PD affected fungal growth and AF synthesis. It is likely that the pH of the culture medium will become more acidic with growth of fungi, and we investigated this by measuring the pH of the media (Figure 8). Significant differences (*p* < 0.01) in the pH values of PD were observed for the strains *A. parasiticus* NRRL2999, *A. flavus* MAFF111229, *A. flavus* MAFF111259, *A. bombysis* MAFF111712, and *A. nomius* MAFF111739 on addition of carbon. The fungal strains *A. oryzae* RIB40, *A. flavus* IFM55891, and *A. parasiticus* MAFF111256 did not show significant changes in the pH. For YES, carbon addition did not cause significant changes in the pH values for any of the strains.

**Figure 9:**
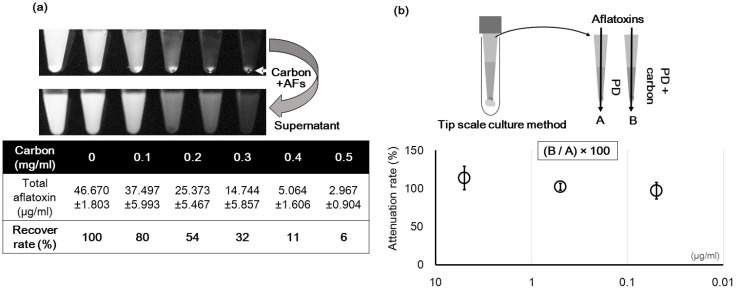
Adsorption of AF by carbon. (**a**) Influence of the carbon concentration on the AF concentration. The fluorescent sediment is AF adsorbed to carbon. The table shows the concentration of AF remaining in the supernatant; (**b**) Recovery of AF from a PD liquid culture containing 0.3 mg/mL carbon (B) compared to that without carbon (A) (*n* = 3).

Additionally, in the liquid culture, carbon could adsorb the AF, and this might reduce the concentration of AF. To investigate this, we observed the adsorption ratios of AF at liquid conditions. In an experiment conducted in a centrifugation tube, 0.3 mg/mL carbon was mixed with an AF standard dissolved in distilled water. This reduced the AF concentration from approximately 46.67 μg/mL to 14.74 μg/mL (Figure 9a). Although addition of carbon to the PD liquid culture affected the recovery of AF (Figure 9b), this difference was not significant. This result was consistent at other AF concentrations. 

## 3. Discussion

### 3.1. Altering the Culture Medium to Enhance the Fluorescence Intensity

Culture media containing CD have been used to detect AF synthesis in the past [14,15,16,17,18]. However, in some cases, detection of AF synthetic fungi with this method is not reliable. In our study, extremely weak fluorescent signals were obtained for *A. flavus* IFM55891 and *A. bombysis* MAFF111712, and these signals were not sufficient for detecting AF synthesis. To improve the signal intensity, we added 0.3 mg/mL carbon to the culture medium. The primary purpose of the carbon was to attenuate reflection of light from the surface of culture plate so that the fluorescence signal would be more visible. The carbon reduced light reflection by approximately 30% compared to the plate without carbon (Figure 1c). Therefore, carbon addition is a useful method for increasing the visibility of AF-derived fluorescence. Another technical problem is that this method is frequently conducted under the transmitted UV light conditions even though the wavelength is not suitable for optical detection. In the present study, reflected and transmitted UV light were compared (Figure 1b). The reflected light shifted to a longer wavelength, this implies that the observation of fluorescence will be easier than it was initially, and this could contribute to the improved visibility of fluorescence. Taken together, the results suggest that addition of carbon and CD to the medium and observation of reflected light are the preferred conditions.

Addition of carbon to the PDA greatly improved the detection efficiency, and its addition to YES also improved the visibility of fluorescence (Figure 2). Among the different CDs, αCD showed the strongest fluorescent intensity (Figure 3), which indicates that it is the most appropriate CD for detecting AF synthetic fungi. However, addition of both αCD and carbon to the PDA induced very strong fluorescence, and this obscured the fluorescence color occasionally (Figure 4). This made it difficult to determine the *Aspergillus* species using the color of fluorescence. The *A. flavus* strains showed bluish fluorescence, whereas the fluorescence from the other AF synthetic fungi was whiter. The blue color could be attributed to the production of B-type AF (blue fluorescence) by the *A. flavus* strains, and G-type AF (green fluorescence) and B-type AF by the other strains. To date, these differences in fluorescence with *Aspergillus* species have not received as much attention as the differences caused by the media composition or type of CD. Because of the high contrast for the fluorescence, both B- and BG-type could be discriminated relatively easily in this study. The *A. nomius* strain and *A. bombysis* strain also showed BG-type colors for fluorescence, although the colors were slightly different to that observed for *A. parasiticus* strains. This knowledge of the relationship between fluorescence color and fungal strain could be used to determine the *Aspergillus* species. In our experiments, addition of βCD and mβCD resulted in relatively mild fluorescence (Figure 3), and these CDs are probably more suitable for this application than αCD. The fluorescence contrast obtained with mβCD was slightly higher than that obtained with βCD, perhaps because the methyl groups decrease the space within the CD ring. Therefore, mβCD is a better candidate than βCD for addition to culture media to detect AF synthesis.

On the YES medium containing both carbon and αCD, the colony colors were easy to discriminate for the different strains but the shapes were slightly blurred. Therefore, the YES medium could be used to confirm differences in the fluorescence colors by optimizing the medium composition or the incubation conditions. The fluorescent enhancement obtained with αCD, βCD, and mβCD improved when more carbon was added to the medium, and this technique will be useful for further development of the CD method for discrimination of AF synthetic fungi from non-toxigenic strains.

### 3.2. Effect of the Increased AF Volume Involved in Fungal Growth

For the liquid culture conducted in a pipette tip, slow growth was observed for the fungal strain in PD, and incubation for seven days did not show clear colony formation on the liquid surface (Figure 5). With addition of carbon to the PD, a colony formed rapidly, which suggests that carbon affects fungal growth. In the YES liquid culture, rapid growth and colony formation were observed after only four days of incubation. Consequently, the YES liquid culture is better for confirming the presence of AF synthetic fungi than the PD liquid culture. However, because of the rapid growth, the changes observed in the fungal masses on addition of carbon to the YES liquid culture were not significant (*p* < 0.05) for any of the strains, except for *A. bombysis* MAFF111712. The PD-based incubation test showed sufficient differences in both the plate and liquid culture tests, which indicates that addition of carbon is suitable for all PD-based studies.

In this study, all of the strains, except for *A. oryzae* RIB40 and *A. flavus* MAFF111259, cultured in PD with added carbon showed increased AF synthesis regardless of the pH. The relationship between the pH of the PD medium and AF concentrations suggests that the pH is not related to the increase in AF synthesis observed on addition of carbon to the medium.

Carbon can reduce the effect of the acute toxicity of AF [31,32,33,35] by adsorbing it. In the culture plate, carbon was fixed, and may not affect the AF in this way. However, in the liquid culture condition with PD, addition of carbon did not greatly increase adsorption of AF (Figure 9b). That is, the recovery test results suggest that the AF concentration in PD is not greatly influenced by the addition of carbon. This means that the enhancement of fluorescence obtained with addition of carbon does not have any drawbacks in this case.

## 4. Conclusions

Addition of carbon to the culture medium improves the efficiency of optical observation but also facilitates the fungal growth and increases AF synthesis. This method uses only a few reagents and UV light, and does not use any harmful reagents or expensive equipment. Therefore, this culture method with carbon and CD will be useful for detecting AF synthetic fungi in many situations. The mechanism by which carbon increases AF synthesis is not clear. However, further studies on the influence of addition of carbon to the culture medium could be used to develop a technique to regulate AF synthesis in future.

## 5. Materials and Methods

### 5.1. Fungal Species

The following *Aspergillus* strains were obtained: *A. oryzae* RIB40 (NBRC 100959, JCM13832) from the National Research Institute of Brewing (Hiroshima, Japan); *A. flavus* IFM55891 (NBRC 33021, ATC C22546) from Chiba University’s Medical Mycology Research Center (Chiba, Japan); *A. parasiticus* NRRL2999 from Prof. Kimiko Yabe at the Faculty of Environmental and Information Science, Fukui University of Technology (Fukui, Japan); and *A. flavus* MAFF111229, *A. parasiticus* MAFF111256, *A. bombysis* MAFF111712, and *A. nomius* MAFF111739 from the Genebank Project, National Agriculture and Food Research Organization (Ibaraki, Japan). Each sample was cultured on a PDA (Merck, Darmstadt, Germany) slant, and incubated at 25 °C for several days. Spores were collected using 0.05% Tween 80 (ICN Biomedicals Inc, Costa Mesa, CA, USA), and stored at 4 °C.

### 5.2. Culture Media Conditions, and Measurement of Fluorescence

PDA and YES (2% yeast extract (Becton, Dickinson and Company, Franklin Lakes, NJ, USA), 15% sucrose (Wako Pure Chemical Industries, Osaka, Japan), and 1.5% agar (Wako Pure Chemical Industries)) were prepared. Then, 3 mg/mL αCD (TCI, Tokyo, Japan), βCD, mβCD, and γCD (Wako Pure Chemical Industries) were added into each medium. PD broth (Merck) and YES broth without CD were also prepared. Activated charcoal powder (Neutral, Wako Pure Chemical Industries) was added into each medium with the final concentration set at 0.3 mg/mL. Five microliters of each spore solution was dispensed onto the culture plate, and it was incubated at 25 °C for a few days. Then, the culture plates were irradiated with UV light at 365 nm (UVGL-58, UVP, Upland, CA, USA), and the fluorescence signals were captured by a digital camera set to the manual white balance setting (Caplio R3, Ricoh, Tokyo, Japan). Three *Aspergillus* strains (*A. flavus* IFM55891, *A. parasiticus* NRRL2999, and *A. bombysis* MAFFF111712) were incubated on the culture plates. The fluorescence intensities were extracted with digital image analysis software (ImageJ, National Institutes of Health [36]). The change in the fluorescence intensity was calculated from the results for triplicate samples. For the measurement of the recovery rate of AF, serial dilutions of AF and carbon were used. The concentration range for diluted AF was 0.05 to 5 μg/mL.

### 5.3. Measurement of Light Attenuation under Reflected and Transmitted Light Conditions

For reflected light, the culture plate was turned upside down and irradiated with UV light from the top and the side. The wavelength of reflected light (360 to 420 nm) was measured using a spectroradiometer (CL-500A, KONICA MINOLTA, Tokyo, Japan).

For transmitted light, the culture plate was irradiated with an UV transilluminator (MLB-16, MAESTROGEN, Las Vegas, NV, USA) from below, and the wavelength of reflected light (360 to 420 nm) was measured with the spectroradiometer placed above the plate. The PFD (μmol·m^–2^·s^–1^) of visible light was also calculated using a program (CL-S10w, Konica Minolta, Tokyo, Japan) with the spectroradiometer.

Blank experiments were conducted without the culture plate. Each measurement was conducted more than four times, and the averages were calculated.

### 5.4. Small-Scale Liquid Culture Conditions for AF Synthesis

A small-scale culture method using a micropipette tip [34] was conducted in this study. Culture medium (295 µL) was dispensed into a 1-mL tip, the end of which was pre-stuffed with glass wool and sealed using parafilm. Then, 5 μL of a spore solution was added to the tip (Figure 9b). After covering with an aluminum cap, the culture tip was incubated at 25 °C for 4 or 7 days. The parafilm was removed from the culture tip and it was placed in a glass microtube, which was centrifuged at 400× *g* for 1 min (EX-126, TOMY Digital Biology, Tokyo, Japan) and the supernatant was collected. Standard samples of the culture medium and AF (Wako Pure Chemical Industries) were also dispensed into the culture tip and then collected by centrifugation. The culture tip without medium was weighed, and the masses before and after were used to monitor mycelia growth. To measure the pH of the media, aliquots of the culture media (50 μL) and distilled water (100 μL) were added to the wells in a 96 well round-bottom plate. Microscale electrodes (HI1093B, Hanna Instruments, Woonsocket, RI, USA) connected to a pH meter (AB15, Thermo Fisher Scientific, Waltham, MA, USA) were inserted into each well to measure the pH.

### 5.5. High Performance Liquid Chromatography

Culture medium and an equivalent volume of chloroform were dispensed into a new microtube, and vortex mixed for 10 s. The chloroform was collected and transferred to a new microtube, and then reduced to dryness in a draft chamber. An aliquot of trifluoroacetic acid (Wako Pure Chemical Industries) equal to 0.1 times of the volume of the chloroform was added to the microtube, followed by vortex mixing for 5 s. After incubation for more than 10 min, a solution of acetonitrile in distilled water (10:90, *v*/*v*) was added to the microtube, with the volume equal to 0.9 times the volume of chloroform. An aliquot (20 µL) of the sample solution was injected into a high performance liquid chromatography system (SCL-10A, Shimadzu, Kyoto, Japan) with a fluorescence detector (λ_Ex_ = 365 nm, λ_Em_ = 455 nm; RF-535, Shimadzu). The mobile phase was a mixture of distilled water:methanol:acetonitrile (60:30:10, *v*/*v*/*v*) at a flow rate of 1 mL/min.

## Figures and Tables

**Table 1:** AF concentrations obtained with carbon addition to the PD, and increases (%) compared to the PD without carbon.

AF Type	AFB_1_	AFB_2_	AFG_1_	AFG_2_	Total
AF concentrations (μg/mg) ± S.D. ^1^					
F1	0.135 ± 0.048	0.002 ± 0.001	-	-	0.137 ± 0.048
F1 + C ^2^	0.194 ± 0.072	0.008 ± 0.003	-	-	0.202 ± 0.075
F2	0.552 ± 0.133	0.001 ± 0.001	-	-	0.553 ± 0.134
F2 + C	3.029 ± 0.468	0.015 ± 0.003	0.008 ± 0.003	0.020 ± 0.005	3.072 ± 0.467
P1	0.090 ± 0.012	0.005 ± 0.000	0.279 ± 0.057	0.006 ± 0.001	0.379 ± 0.069
P1 + C	0.176 ± 0.024	0.014 ± 0.002	1.117 ± 0.187	0.050 ± 0.007	1.357 ± 0.213
P2	0.104 ± 0.009	0.008 ± 0.001	0.101 ± 0.009	-	0.212 ± 0.018
P2 + C	0.076 ± 0.016	0.009 ± 0.002	0.426 ± 0.050	0.025 ± 0.004	0.537 ± 0.071
N	0.263 ± 0.042	0.001 ± 0.001	0.007 ± 0.003	-	0.271 ± 0.045
N + C	0.578 ± 0.093	0.002 ± 0.000	0.108 ± 0.020	-	0.688 ± 0.108
B	0.130 ± 0.022	0.002 ± 0.001	0.110 ± 0.018	-	0.243 ± 0.040
B + C	0.816 ± 0.069	0.023 ± 0.002	1.203 ± 0.099	0.013 ± 0.002	2.054 ± 0.168
Changes of AF concentrations caused by carbon supply (%)					
F1 + C	144	409	-	-	148
F2 + C	548	2422	-	-	555
P1 + C	195	286	401	891	358
P2 + C	74	121	424	5342	253
N + C	220	276	1501	-	254
B + C	627	931	1090	-	845

^1^ Standard deviation, ^2^ 0.3 mg/mL carbon, F1 = *A. flavus* IFM55891, F2 = *A. flavus* MAFF111229, P1 = *A. parasiticus* NRRL2999, P2 = *A. parasiticus* MAFF111256, N = *A. nomius* MAFF111739, B = *A. bombysis* MAFF111712 (*n* = 4).

**Table 2:** AF concentrations obtained with carbon addition to the YES, and increases (%) compared to the YES without carbon.

AF type	AFB_1_	AFB_2_	AFG_1_	AFG_2_	Total
AF concentrations (μg/mg) ± S.D. ^1^					
F1	0.522 ± 0.090	0.006 ± 0.003	-	-	0.528 ± 0.092
F1 + C ^2^	0.619 ± 0.146	0.011 ± 0.005	-	-	0.629 ± 0.150
F2	1.503 ± 0.098	0.005 ± 0.000	0.005 ± 0.001	0.002 ± 0.000	1.516 ± 0.098
F2 + C	1.113 ± 0.094	0.004 ± 0.000	0.006 ± 0.002	0.003 ± 0.000	1.126 ± 0.096
P1	0.137 ± 0.013	0.002 ± 0.001	0.981 ± 0.050	0.033 ± 0.004	1.153 ± 0.065
P1 + C	0.100 ± 0.010	0.001 ± 0.001	0.985 ± 0.057	0.039 ± 0.003	1.125 ± 0.070
P2	0.200 ± 0.043	0.010 ± 0.002	0.377 ± 0.101	0.018 ± 0.005	0.606 ± 0.149
P2 + C	0.206 ± 0.049	0.011 ± 0.003	0.557 ± 0.104	0.032 ± 0.009	0.806 ± 0.158
N	2.141 ± 0.182	0.029 ± 0.001	0.730 ± 0.059	0.012 ± 0.002	2.912 ± 0.238
N + C	1.934 ± 0.117	0.025 ± 0.009	0.747 ± 0.104	0.011 ± 0.001	2.717 ± 0.165
B	1.565 ± 0.214	0.036 ± 0.006	2.444 ± 0.257	0.039 ± 0.005	4.085 ± 0.478
B + C	1.405 ± 0.272	0.033 ± 0.007	2.118 ± 0.236	0.035 ± 0.006	3.592 ± 0.516
Changes of AF concentrations caused by carbon supply (%)					
F1 + C	118.5	174.2	-	-	119.1
F2 + C	74.1	75.7	117.6	139.0	74.3
P1 + C	73.1	45.3	100.4	117.9	97.6
P2 + C	102.7	111.5	147.7	177.6	133.1
N + C	90.3	87.4	102.3	85.8	93.3
B + C	89.8	89.9	86.7	90.8	87.9

^1^ Standard deviation, ^2^ 0.3 mg/mL carbon, F1 = *A. flavus* IFM55891, F2 = *A. flavus* MAFF111229, P1 = *A. parasiticus* NRRL2999, P2 = *A. parasiticus* MAFF111256, N = *A. nomius* MAFF111739, B = *A. bombysis* MAFF111712 (*n* = 4).
